# Urethane Synthesis in the Presence of Organic Acid Catalysts—A Computational Study

**DOI:** 10.3390/molecules29102375

**Published:** 2024-05-17

**Authors:** Hadeer Q. Waleed, Béla Viskolcz, Béla Fiser

**Affiliations:** 1 Institute of Chemistry, University of Miskolc, 3515 Miskolc-Egyetemváros, Hungary; 2 Higher Education and Industrial Cooperation Centre, University of Miskolc, 3515 Miskolc-Egyetemváros, Hungary; 3 Ferenc Rakoczi II Transcarpathian Hungarian College of Higher Education, 90200 Beregszász, Transcarpathia, Ukraine; 4 Department of Physical Chemistry, Faculty of Chemistry, University of Lodz, 90-236 Lodz, Poland

**Keywords:** composite method, acid catalysts, polyurethane, polymers, materials

## Abstract

A general mechanism for catalytic urethane formation in the presence of acid catalysts, dimethyl hydrogen phosphate (DMHP), methanesulfonic acid (MSA), and trifluoromethanesulfonic acid (TFMSA), has been studied using theoretical methods. The reaction of phenyl isocyanate (PhNCO) and butan-1-ol (BuOH) has been selected to describe the energetic and structural features of the catalyst-free urethane formation. The catalytic activities of DMHP, MSA, and TFMSA have been compared by adding them to the PhNCO–BuOH model system. The thermodynamic properties of the reactions were computed by using the G3MP2BHandHLYP composite method. It was revealed that in the presence of trifluoromethanesulfonic acid, the activation energy was the lowest within the studied set of catalysts. The achieved results indicate that acids can be successfully employed in urethane synthesis and the mechanism was described.

## 1. Introduction

**Figure 1:**
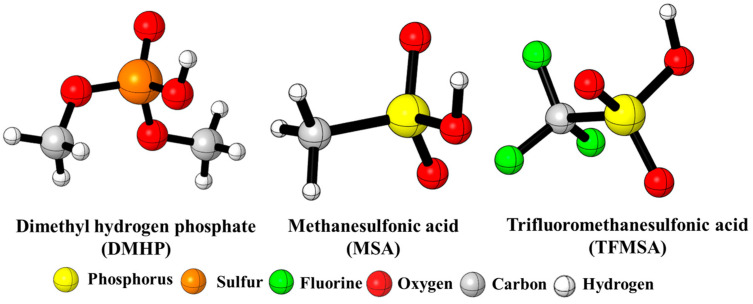
Three-dimensional structures of the studied catalysts.

The field of polymer science emerged to develop new materials for growing civil and military needs. It tends to be more interdisciplinary than most sciences, combining chemistry, chemical engineering, and other fields as well [1,2]. In 1937, one of the most special polymer types with versatile properties was discovered [3]. This special type of polymer is polyurethane (PU), which was developed by Otto Bayer to compete with nylon [4,5]. Bayer’s invention ranks among the most important breakthroughs in polymer science. At the beginning of the 1950s, researchers were able to use PUs to produce soft foam plastic. In the early 1960s, synthetic PU adhesive, PU flexible fiber, and other types of PUs were developed [6]. From the mid-1960s to the 1990s, the development of polyurethanes significantly increased and they became unavoidable in many applications [7,8]. In 2018, the PU market reached USD 59.5 billion globally, and it is expected to grow between 2019 and 2026 by 5.8% CAGR (compound annual growth rate) [9,10]. Polyurethane is used in a large array of industries as flexible and rigid foams, elastomers, and thermoplastic materials [11]. Most of the PU types are designed to make life more comfortable and products more durable [12,13]. Polyurethanes (PUs) are a special group of heterochain polymers formed by the reaction of isocyanate (NCO) and hydroxyl (OH) groups [14,15]. Isocyanate is a chemical that contains at least one isocyanate group (-N=C=O) in its structure. In PU synthesis, two types of isocyanates, aromatic and aliphatic ones, are used [16]. The other main raw materials in PU synthesis are polyols containing two or more hydroxyl groups [17]. In addition to the effect of the chemical structure and the functionality of isocyanates and polyols on urethane formation [18], polyurethane synthesis can be finetuned by applying various additional compounds such as catalysts, chain extenders, crosslinkers, surfactants, and blowing agents [19]. In relation to PU synthesis, catalysts are often used to accelerate the reaction rate of polynucleophiles with isocyanate groups or to promote the trimerization of the isocyanate group to form crosslinked polymers. In the production of PUs, the amount of applied catalysts is small, but their impact is significant [20]. Catalysts play an important role in the control and balance between the gelling and blowing reactions. They help to accurately control the relative reaction rates of the isocyanate with both alcohol and water. The imbalance between these reactions is one of the reasons for the collapse of foam or the formation of inappropriate cells that can be closed or opened prematurely [21,22]. Polyurethane catalysts mainly include organic acids, organic bases (amine catalysts), and organo-tin (organometallic) compounds [23,24,25,26]. Organic acid catalysts are a type of organic catalysts which show significant efficiency in urethane formation (alcohol−isocyanate) reactions [27]. The use of acid catalysis is expected to expand the range of metal-free polyurethane syntheses under both solution and bulk polymerization conditions [27]. Meanwhile, there are certain organic acids which are able to promote urethane formation under mild polymerization conditions and low catalyst loadings [28]. On the other hand, organic acid catalysts can extend the range of polymerizable monomers that have amides or additional functionalities that are sensitive to base catalysis [29]. The effect of organic acids on urethane formation has been investigated and the reaction between isocyanates and alcohols in the presence of these catalysts was studied at high temperatures. It was found that organic acids in certain aspects are more efficient in activating isocyanates than tin-based catalysts. Previously, the effect of amine catalysts on urethane formation using the phenyl isocyanate (PhNCO) -- butan-1-ol (BuOH) model reaction and the G3MP2BHandHLYP composite method was investigated. The accuracy of the method was validated by conducting kinetic experiments, and the theoretical results were in good agreement with the experimental ones. These results prove the validity of the proposed mechanism and verify the method selection as well [30,31,32,33,34,35,36]. Herein, the reaction between PhNCO and BuOH is studied in the presence of acid catalysts, dimethyl hydrogen phosphate (DMHP), methanesulfonic acid (MSA), and trifluoromethanesulfonic acid (TFMSA) (Figure 1). Dimethyl hydrogen phosphate is an organophosphorus compound. It is a colorless, odorless liquid that is miscible with water and many organic solvents. DMHP is versatile, making it valuable in various industrial and research settings with applications in chemical synthesis, catalysis, analytical chemistry, electrochemistry, and surfactant technology. Its ability to introduce phosphate groups into organic molecules makes it applicable in diverse areas [37,38], while methanesulfonic acid is a strong organic acid that is highly soluble in water and miscible with many organic solvents. This solubility makes it convenient for use in reactions. MSA is commonly used as a catalyst and acid promoter in organic synthesis reactions. It can facilitate a variety of reactions. Its strong acidity and compatibility with a wide range of substrates make it a versatile tool in synthetic chemistry [39,40]. Trifluoromethanesulfonic acid has several applications across various fields due to its strong acidity. TFMSA is widely used as a strong acid catalyst in various organic synthesis reactions. Its strong acidity promotes reaction rates and facilitates the formation of the desired products. TFMSA is utilized as a catalyst in different polymerization reactions, especially in the synthesis of polymers and copolymers. It can initiate polymerization reactions and control kinetics and molecular weight distributions [41,42]. Thus, these species can also be effective in urethane synthesis. To study the thermodynamic properties and understand the reactions from a mechanistic point of view, computational tools have been used.

## 2. Results and Discussion

**Figure 2:**
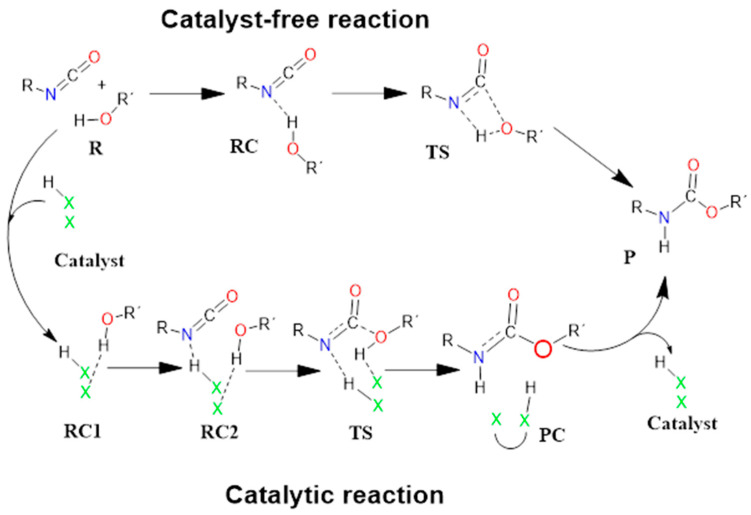
Schematic representation of the proposed general reaction mechanism of isocyanates and alcohols in the absence (upper row) and presence (bottom) of acid catalysts. RC—reactant complex; TS—transition state; PC—product complex; and P—product.

The recently studied catalyst-free reaction mechanism was utilized as a reference [30,32,33,34] (Figure 2). The reaction between butan-1-ol (BuOH) and phenyl isocyanate (PhNCO) was selected as a model to describe the energetic and structural features of catalyst-free urethane formation. For the catalyst-free system, the corresponding thermodynamic properties were computed (Table 1).

**Figure 3:**
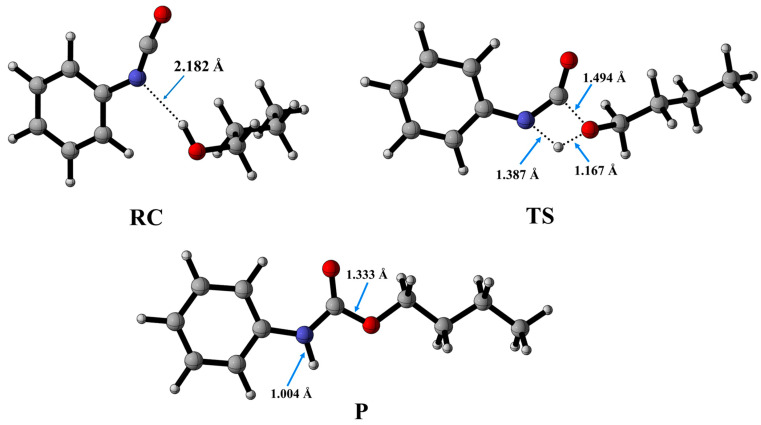
Optimized structures along the reaction pathway between phenyl isocyanate and butan-1-ol calculated at the BHandHLYP/6-31G(d) level of theory in acetonitrile at 298.15 K and 1 atm. RC—reactant complex; TS—transition state; P—product.

From the optimized geometries (Figure 3), it can be observed that the urethane bond formed via a concerted mechanism.

**Figure 4:**
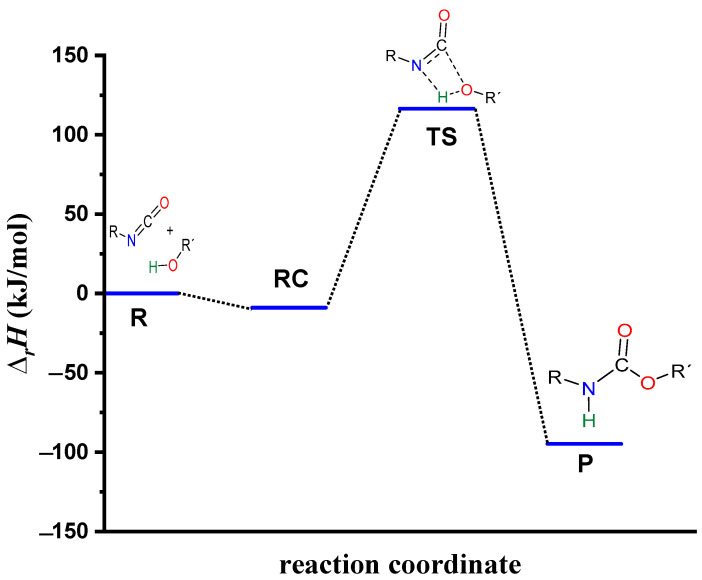
Energy profile (relative enthalpy (∆_r_*H*)) of the catalyst-free phenyl isocyanate and butan-1-ol reaction calculated using the G3MP2BHandHLYP composite method in acetonitrile, using the SMD implicit solvent model at 298.15 K and 1 atm.

The process begins with the formation of the reactant complex (RC, PhNCO--BuOH) with a N-H distance of 2.182 Å, and the corresponding relative enthalpy is −8.97 (kJ/mol) (Figure 3). Following the reactant complex, a transition state (TS) is formed, within which a bond will form between the oxygen of the butan-1-ol and the carbon of the isocyanate group. The C-O distance in the TS is 1.494 Å. Additionally, hydrogen is donated from the butan-1-ol’s hydroxyl group to the isocyanate’s nitrogen, the N-H distance is decreased to 1.387 Å, and the relative enthalpy of the TS is 116.49 kJ/mol. Consequently, the final product (P) is achieved through the transition state, and the corresponding relative enthalpy is −94.84 kJ/mol (Figure 4).

**Figure 5:**
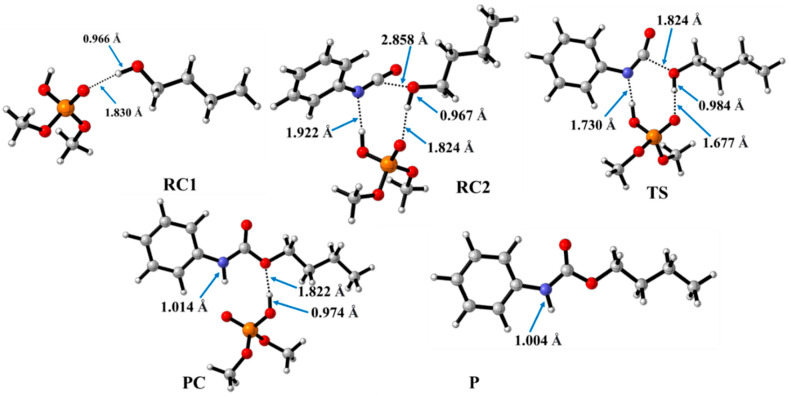
Optimized structures along the reaction pathway between phenyl isocyanate and butan-1-ol in the presence of dimethyl hydrogen phosphate (DMHP) calculated at the BHandHLYP/6-31G(d) level of theory (298.15 K and 1 atm) in acetonitrile. RC—reactant complex; TS—transition state; PC—product complex; and P—product.

**Figure 6:**
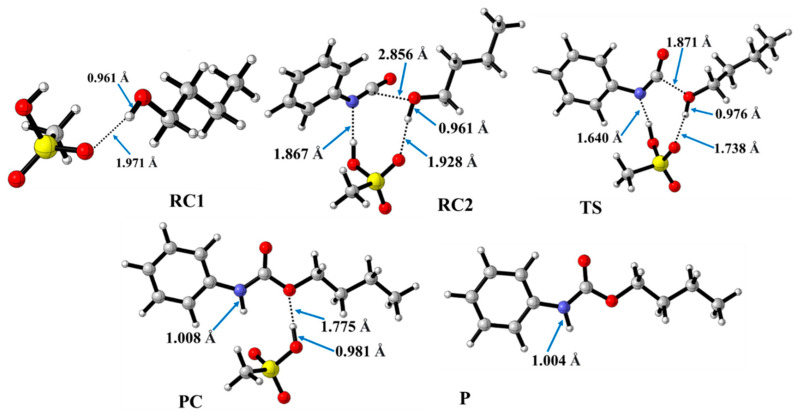
Optimized structures along the reaction pathway between phenyl isocyanate and butan-1-ol in the presence of methanesulfonic acid (MSA) calculated at the BHandHLYP/6-31G(d) level of theory (298.15 K and 1 atm) in acetonitrile. RC—reactant complex; TS—transition state; PC—product complex; and P—product.

**Figure 7:**
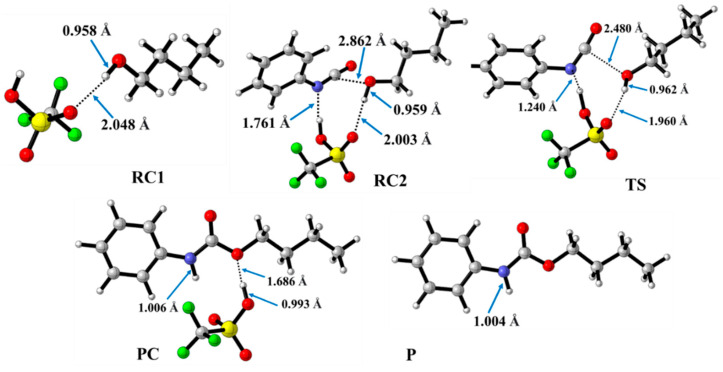
Optimized structures along the reaction pathway between phenyl isocyanate and butan-1-ol in the presence of trifluoromethanesulfonic acid (TFMSA) calculated at the BHandHLYP/6-31G(d) level of theory (298.15 K and 1 atm) in acetonitrile. RC—reactant complex; TS—transition state; PC—product complex; and P—product.

In contrast to the catalyst-free case, urethane formation in the presence of acid catalysts includes five steps (Figure 2). First, a complex (RC1) between the alcohol and the catalyst forms, while the distance between the catalyst’s oxygen and the hydroxyl hydrogen of butan-1-ol is in the range of 1.830 and 2.048 Å (Figure 5, Figure 6 and Figure 7) (Table 2, O-H*). This is supposed to mimic the industrial urethane synthesis, within which the catalyst is first mixed into the polyol. Then, in the next step, the isocyanate, in the current case PhNCO, is added to the system, and RC2, a trimolecular complex, is formed. In this step, a new interaction occurs between the butan-1-ol’s oxygen and the isocyanate group, while only insignificant changes can be identified in the length of the previously established O-H*.

**Figure 8:**
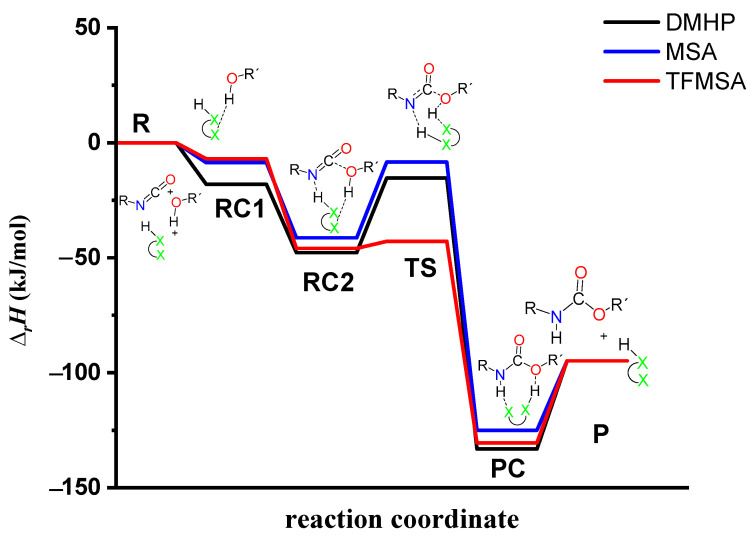
Relative enthalpy (∆rH) profile of the studied catalyzed urethane formation reactions in the presence of dimethyl hydrogen phosphate (DMHP), methanesulfonic acid (MSA), and trifluoromethanesulfonic acid (TFMSA) calculated at the G3PMP2BHandHLYP level of theory (298.15 K and 1 atm) in acetonitrile using the SMD implicit solvent model, respectively.

The effect on the O-H** bond length is even smaller and almost no change is observed between RC1 and RC2 (Table 2). The most stable butan-1-ol–catalyst and trimolecular complexes are formed in the case of DMHP (∆*_r_H* = −18.09 for RC1, and RC2, ∆_r_*H* = −47.79 kJ/mol). Meanwhile, the TFMSA–butan-1-ol complex is the least stable bimolecular complex (∆*_r_H* = −6.94 kJ/mol), while the MSA–butan-1-ol-PhNCO complex is the least stable trimolecular complex (RC2, ∆*_r_H* = −41.30 kJ/mol) (Table 1 and Figure 8).

In the next step, a transition state develops in the presence of the catalyst, where a proton transfer occurs from the hydroxyl group of butan-1-ol to the oxygen of the acid catalyst, resulting in a decrease in the O-H* distance, which ranges from 1.677 to 1.960 Å. Meanwhile, protons will be donated from the acid catalyst to the nitrogen of the isocyanate, with N-H distances ranging from 1.240 to 1.730 Å. Furthermore, a new bond will form between the carbon of the isocyanate group and the oxygen of butan-1-ol, significantly reducing the distances to 1.824–2.480 Å. At the same time, the O-H** distance increased. It was noticed that the C-O distance of TS is large for TFMSA compared to other catalysts. The variation in interatomic distances can be linked to the proton affinity, electronic structure, and charge distribution of the acid. The potential energy curve shows that the relative enthalpy of the transition state is lowest (−42.85 kJ/mol) when trifluoromethanesulfonic acid is considered. In contrast, with dimethyl hydrogen phosphate and methanesulfonic acid, there are increases of ~27 kJ/mol and 51 kJ/mol, respectively **(**Figure 8). The results showed that the barrier height of the reaction in the presence of acid catalysts compared to the catalytic-free system (∆∆_r_*H* = ∆_r_*H_Cat.-free_*_(RC-TS)_ − ∆_r_*H_Cat._*_(RC1-TS)_) significantly decreased by 127.7, 125.3, and 89.6 kJ/mol for DMHP, MSA, and TFMSA, respectively (Table 1, and Figure 8). It must be noted that the barrier height is computed as the enthalpy difference between the RC1 and TS, as the first step in the experiment is to mix the catalyst into the alcohol.

Before the reaction completes, a product complex (PC) forms where a bond is formed between the carbon of the isocyanate group and the oxygen of the butanol. Additionally, an N-H bond forms with distances ranging from 1.006 to 1.014 Å, and a PC relative enthalpy range of −125.01 and −133.12 kJ/mol. At this point, the catalyst is released, and the urethane bond is already complete (Figure 5, Figure 6 and Figure 7). In the final step, the catalysts and the product are separated (P), with the corresponding relative enthalpy of −94.84 kJ/mol where both the strength of a given acid and the nucleophilicity of its conjugate base play a vital role in the bifunctional catalysis of urethane formation. Therefore, along with the whole reaction mechanism, the trifluoromethanesulfonic acid (TFMSA) catalyst was the most effective and provided the most favorable pathway.

## 3. Methods

The molecules were optimized by using the BHandHLYP (Becke, Half-and-Half, Lee–Yang–Parr) [43] density functional method in combination with the 6-31G(d) [44,45,46] basis set. The effect of the solvent (e.g., acetonitrile, MeCN, εr = 35.688) was also considered by employing the SMD polarizable continuum model [47]. Several different density functional theory methods such as B3LYP [48], BHandHLYP [43], and ωB97X-D [49], in combination with the 6-31G(d) [44,45,46] basis set, were tested to investigate urethane formation using organic catalysts. However, only the BHandHLYP method was able to identify all the critical points on the potential energy surfaces (PESs) of the studied catalytic processes, proving its efficiency in studying catalytic urethane formation reactions [30,34,35,50]. Furthermore, to determine the thermodynamic properties of the studied system and to verify the nature of the stationary points on the potential energy surface, frequency calculations were performed. Meanwhile, to further improve the accuracy of the results, the G3MP2BHandHLYP composite method [50,51,52] was applied. On each optimized structures, two separate single-point energy calculations were performed at the QCISD(T)/6-31G(d) and MP2/GTMP2Large levels of theory and the previously determined composite scheme was applied [53,54].

All the calculations were carried out using the Gaussian 09 program [55]. The geometric configurations in this study were all displayed by using the CYLview program [56].

## 4. Conclusions

Urethane formation in organic acid-catalyzed processes was studied using computational chemical tools, including both density functional theory (BHandHLYP/6-31G(d)) and composite methods (G3MP2BHandHLYP). A general mechanism for catalytic urethane formation in the presence of three different acid catalysts, dimethyl hydrogen phosphate (DMHP), methanesulfonic acid (MSA), and trifluoromethanesulfonic acid (TFMSA), has been examined and described. The reaction mechanism of acid-catalyzed urethane formation contains five steps, which include one transition state and product complex. This route is different from the mechanism for catalytic urethane formation in the presence of amine catalysts. Meanwhile, it is slightly similar to the catalyst-free process as both have one transition state. However, the results showed that the barrier height of the reaction in the presence of acid catalysts compared to the catalyst-free system (∆∆_r_*H* = ∆_r_*H_Cat.-free_*_(RC-TS)_ − ∆_r_*H_Cat._*_(RC1-TS)_) significantly decreased by 127.7, 125.3, and 89.6 kJ/mol for DMHP, MSA, and TFMSA, respectively. It was found that TFMSA was the most potent organic acid catalyst within the studied set of species. This finding can be used to design better candidates for future synthetic explorations.

## Figures and Tables

**Table 1:** The relative enthalpy (∆_r_*H*) of the reaction between phenyl isocyanate and butan-1-ol with and without catalysts, calculated using the G3MP2BHandHLYP composite method (298.15 K and 1 atm) in acetonitrile, using the SMD implicit solvent model. R—reactant; RC—reactant complex; TS—transition state; PC—product complex; and P—product.

∆*_r_H* (kJ/mol)						
	R	RC1	RC2	TS	PC	P
**Catalyst-free system**	0.00	-	−8.97′	116.49	-	−94.84
**DMHP**	0.00	−18.09	−47.79	−15.31	−133.12	−94.84
**MSA**	0.00	−8.66	−41.30	−8.44	−125.01	−94.84
**TFMSA**	0.00	−6.94	−45.94	−42.85	−130.46	−94.84

’RC for catalyst-free reaction.

**Table 2:** N-H, O-H, and C-O bond lengths (Å) along the pathway of the phenyl isocyanate (PhNCO) and butan-1-ol reaction in the presence of the studied catalysts, dimethyl hydrogen phosphate (DMHP), methanesulfonic acid (MSA), and trifluoromethanesulfonic acid (TFMSA), calculated at the BHandHLYP/6-31G(d) level of theory (298.15 K and 1 atm) in acetonitrile. O-H* for catalysts; O-H** for butan-1-ol.

Catalysts		RC1	RC2				TS				PC			P
	O-H*	O-H**	N-H	O-H*	O-H**	C-O	N-H	O-H*	O-H**	C-O	N-H	O-H*	O-H**	N-H
**DMHP**	1.830	0.966	1.922	1.824	0.967	2.858	1.730	1.677	0.984	1.824	1.014	0.974	1.822	1.004
**MSA**	1.971	0.961	1.867	1.928	0.961	2.856	1.640	1.738	0.967	1.871	1.008	0.981	1.775	1.004
**TFMSA**	2.048	0.958	1.761	2.003	0.959	2.862	1.240	1.960	0.962	2.480	1.006	0.993	1.686	1.004

## Data Availability

The structures, figures, and additional tables are available in the Supplementary Materials.

## Supplementary Materials

The following supporting information can be downloaded at: https://www.mdpi.com/article/10.3390/molecules29102375/s1, Tables S1 and S2: Thermodynamic properties calculated at the BHandHLYP/6-31G(d) and G3MP2BHandHLYP levels of theory. Table S3: Cartesian coordinates of the stationary points for the studied species.
