# Supplementary material for: Urethane Synthesis in the Presence of Organic Acid Catalysts—A Computational Study
Source: Molecules. 2024 May 17;29(10):2375. doi: 10.3390/molecules29102375 (PMC11123846; doi:10.3390/molecules29102375)
Supplement: Supplementary file 1 [file molecules-29-02375-s001.zip › molecules-2996001-supplementary.pdf]

molecules

Urethane Synthesis in the Presence of Organic Acid Catalysts - A Computational Study

Hadeer Q. Waleed [1,2], Béla Viskolcz [1], and Béla Fiser [2,3,4,*]

[1] Institute of Chemistry, University of Miskolc, 3515 Miskolc-Egyetemváros, Hungary
[2] Higher Education and Industrial Cooperation Centre, University of Miskolc, 3515 Miskolc-Egyetemváros,Hungary
[3] Ferenc Rakoczi II Transcarpathian Hungarian College of Higher Education, 90200 Beregszász, Transcarpathia, Ukraine
[4] Department of Physical Chemistry, Faculty of Chemistry, University of Lodz, Lodz, Poland
* Correspondence: bela.fiser@uni-miskolc.hu (B.F.)

Supporting Information

Table S1. Zero-point corrected relative energies ($\Delta_r E_0$), relative enthalpies ($\Delta_r H$), relative Gibbs free energies ($\Delta_r G$), and relative entropies ($\Delta_r S$) of the reaction between phenyl isocyanate and butan-1-ol in presence of the studied catalysts, dimethyl hydrogen phosphate (DMHP), methanesulfonic acid (MSA), and trifluoromethanesulfonic acid (TFMSA) calculated at the BHandHLYP/6-31G(d) level of theory in acetonitrile using the SMD implicit solvent model at 298.15 K and 1 atm. R – reactant, RC – reactant complex, TS – transition state, IM – intermediate, PC – product complex, P – product.

	**$\Delta_r E_0$ (kJ/mol)**					
	**R**	**RC1**	**RC2**	**TS**	**PC**	**P**
**Cat.-free**	0.0	-	-10.48′	122.75	-	-120.94
**DMHP**	0.0	-21.67	-41.77	-7.93	-151.94	-120.94
**MSA**	0.0	-13.19	-34.19	0.53	-144.67	-120.94
**TFMSA**	0.0	-9.95	-35.97	-25.77	-146.28	-120.94
	**$\Delta_r H$ (kJ/mol)**					
	**R**	**RC1**	**RC2**	**TS**	**PC**	**P**
**Cat.-free**	0.0	-	-8.23′	120.13	-	-123.20
**DMHP**	0.0	-19.32	-38.12	-9.40	-153.13	-123.20
**MSA**	0.0	-11.42	-30.82	-1.35	-145.96	-123.20
**TFMSA**	0.0	-7.28	-32.57	-24.36	-147.11	-123.20
	**$\Delta_r G$ (kJ/mol)**					
	**R**	**RC1**	**RC2**	**TS**	**PC**	**P**
**Cat.-free**	0.0	-	29.65′	173.69	-	-69.89
**DMHP**	0.0	13.78	43.57	92.46	-51.18	-69.89
**MSA**	0.0	25.74	52.01	98.52	-45.29	-69.89
**TFMSA**	0.0	30.20	56.17	67.24	-47.89	-69.89
	**$\Delta_r S$ (cal/mol*K)**					
	**R**	**RC1**	**RC2**	**TS**	**PC**	**P**
**Cat.-free**	0.0	-	-30.37′	-42.94	-	-42.73
**DMHP**	0.0	-26.54	-65.49	-81.66	-81.73	-42.73

MSA	0.0	-29.79	-66.40	-80.06	-80.71	-42.73
TFMSA	0.0	-30.04	-71.14	-73.43	-79.54	-42.73

* RC for catalyst-free (cat.-free) reaction.

Table S2. Zero-point corrected relative energies ($\Delta_r E_0$), relative enthalpies ($\Delta_r \boldsymbol{H}$), relative Gibbs free energies ($\Delta_r \boldsymbol{G}$), and relative entropies ($\Delta_r S$) of the reaction between phenyl isocyanate and butan-1-ol in presence of the studied catalysts, dimethyl hydrogen phosphate (DMHP), methanesulfonic acid (MSA), and trifluoromethanesulfonic acid (TFMSA) calculated at the G3MP2BHandHLYP/6-31G(d) level of theory in acetonitrile using the SMD implicit solvent model at 298.15 K and 1 atm. R – reactant, RC – reactant complex, TS – transition state, IM – intermediate, PC – product complex, P – product.

	$\Delta_r E_0$ (kJ/mol)					
	**R**	**RC1**	**RC2**	**TS**	**PC**	**P**
**Cat.-free**	0.0	-	-11.22′	119.11	-	-92.58
**DMHP**	0.0	-20.44	-51.44	-13.84	-131.93	-92.58
**MSA**	0.0	-10.44	-44.68	-6.57	-123.71	-92.58
**TFMSA**	0.0	-9.61	-49.35	-44.25	-129.63	-92.58
	**Δ_rG (kJ/mol)**					
	**R**	**RC1**	**RC2**	**TS**	**PC**	**P**
**Cat.-free**	0.0	-	28.91′	170.05	-	-41.54
**DMHP**	0.0	15.02	33.91	86.56	-31.17	-41.54
**MSA**	0.0	28.50	41.53	91.43	-24.33	-41.54
**TFMSA**	0.0	30.54	42.80	48.75	-31.24	-41.54
	**Δ_rS (cal/mol*K)**					
	**R**	**RC1**	**RC2**	**TS**	**PC**	**P**
**Cat.-free**	0.0	-	-30.36′	-42.94	-	-42.73
**DMHP**	0.0	-26.54	-65.48	-81.66	-81.73	-42.73
**MSA**	0.0	-35.33	-66.40	-80.06	-80.71	-42.73
**TFMSA**	0.0	-35.59	-71.14	-73.43	-79.54	-42.73

′RC for catalyst-free (cat.-free) reaction.

Table S3. Cartesian coordinates of the stationary points for the studied species. The structures calculated at the BHandHLYP/6-31G(d) level of theory in acetonitrile at 298.15 K and 1 atm. Cat.- catalysts, RC – reactant complex, TS – transition state, IM – intermediate, PC – product complex, P – product.

Keywords included in the input files to carry out the calculations on the studied structures:

BHandHLYP/6-31g(d) opt=(tight) freq scrf=(smd,solvent=acetonitrile)
#BHandHLYP/6-31g(d) opt=(calcfc,tight,ts,noeigentest) freq scrf=(smd,solvent=acetonitrile)
MP2/GTMP2large scrf=(smd,solvent=acetonitrile)
QCISD(T)/6-31G(d) scrf=(smd,solvent=acetonitrile)

DMHP				DMHP-RC1			
O	0.54336800	1.64949200	-0.77148500	O	1.64285200	-1.01233300	1.04050700
P	0.02743800	0.45721300	-0.09567600	C	2.36664900	-0.48912000	-0.04469800
O	0.03813000	0.54766500	1.49910600	H	0.72085800	-1.09591200	0.76428700
O	-1.44868800	0.11245900	-0.52508800	H	1.95428400	0.47310500	-0.36178500
O	0.77257000	-0.91233100	-0.34554100	H	2.31150500	-1.15708600	-0.90967900
H	-0.02815900	1.45581000	1.81581300	C	3.81252800	-0.31098600	0.36602100
C	2.15538000	-1.06019100	-0.00669500	H	3.84924400	0.35211100	1.23108000
H	2.44588200	-2.05099000	-0.32994200	H	4.20168300	-1.27538700	0.69445300

H	2.75243300	-0.31656500	-0.52351900	C	4.68620000	0.24576500	-0.74985600
H	2.29026800	-0.96863900	1.06549500	H	4.28448200	1.20459000	-1.07957800
C	-2.16391600	-0.99868100	0.02356300	H	4.63744000	-0.41919900	-1.61287200
H	-3.16994200	-0.93750000	-0.37011100	C	6.13790000	0.42378700	-0.33054300
H	-1.70327300	-1.92990000	-0.28569600	H	6.73991000	0.82236900	-1.14483700
H	-2.19058700	-0.93545200	1.10596200	H	6.22207400	1.11037700	0.51060200
				H	6.57800200	-0.52490600	-0.02678600
				O	-0.99318500	-1.14588900	0.12378500
				P	-2.02154100	-0.09438500	0.13939100
				O	-2.91963900	-0.08432900	1.45488600
				O	-1.39855400	1.33912900	-0.01808500
				O	-3.10175700	-0.13274300	-1.00428000
				H	-2.45359400	-0.41780200	2.23079300
				C	-3.95763300	-1.27074400	-1.17203200
				H	-4.55571600	-1.07385700	-2.05143900
				H	-3.36567700	-2.16671200	-1.32236700
				H	-4.60008900	-1.38515900	-0.30625500
				C	-2.19677300	2.52938400	0.00983100
				H	-1.50449600	3.35890700	-0.04157700
				H	-2.86366900	2.55068700	-0.84416100
				H	-2.76408700	2.58044200	0.93239200
**DMHP-RC2**				**DMHP-TS**			
O	-1.69464100	0.08607100	-0.77171500	O	1.27356800	-1.19447600	-0.01934200
H	-0.83365000	-0.32137800	-0.93951200	C	2.38606300	-1.94520300	-0.50782200
O	0.72735100	-1.23929600	-1.15911300	H	1.25380300	-0.30484200	-0.44096600
O	2.23095500	-0.82368600	0.88495400	H	2.39720900	-1.90657800	-1.59515100
H	1.54399000	-0.18666500	1.15156600	H	2.20821400	-2.97109000	-0.20751300
N	0.25900500	1.21510500	1.43712200	O	1.23866100	1.27210600	-1.01256100
C	-0.78882600	0.77728000	1.84968800	O	-0.46033600	1.48168500	0.87969600
O	-1.70663100	0.27050000	2.34146600	H	-0.69519700	0.54541000	0.63774200
C	0.60327700	2.35837000	0.68858400	C	-0.41690800	-2.02826800	-0.03129400
C	-0.33841700	3.05105900	-0.06364900	C	-5.35299700	-1.43312000	0.14670000
C	1.92866100	2.76903400	0.71271300	C	-4.56489800	-2.54087000	0.42040500
C	0.05599900	4.16837000	-0.78030900	C	-4.74400600	-0.21973900	-0.13853700
H	-1.35608500	2.70148600	-0.10073100	C	-3.36333300	-0.11444900	-0.15044700
C	2.31070700	3.88802000	-0.01007900	C	-2.57240100	-1.22719400	0.12066500
H	2.64679500	2.21904100	1.29819200	C	-3.18212300	-2.44511400	0.41076700
C	1.37747000	4.59230400	-0.75566800	H	-2.58427800	-3.31313400	0.63125000
H	-0.67422800	4.70626500	-1.36301900	H	-5.02518200	-3.48930900	0.64702400
H	3.33979800	4.20762900	0.01293700	H	-5.34307400	0.65031800	-0.35463600
H	1.67701100	5.46251000	-1.31654100	H	-2.89005900	0.82586900	-0.38010000
C	-2.65696900	-0.94161000	-0.72543800	N	-1.16664900	-1.03294800	0.10781500
H	-2.44957300	-1.63014800	0.09730000	O	-0.19459600	-3.17111700	-0.15662900
H	-2.63437400	-1.52828000	-1.64754300	H	-6.42771800	-1.51434800	0.15619100
C	-4.02859600	-0.32883500	-0.54565500	C	3.68255300	-1.42395600	0.07568100
H	-4.21096000	0.36365500	-1.36808100	H	3.80881100	-0.37906800	-0.20780600
H	-4.02757000	0.26299700	0.36989700	H	3.61430800	-1.45468000	1.16252300
C	-5.14041600	-1.36783600	-0.48844400	C	4.88568400	-2.23240300	-0.39436100
H	-4.94549300	-2.06099200	0.33060300	H	4.93216100	-2.21128600	-1.48333200
H	-5.12686700	-1.96273800	-1.40238300	H	4.75004600	-3.27659900	-0.11193800
C	-6.51864200	-0.74870200	-0.30929600	C	6.19681200	-1.71655600	0.17923700

H	-6.75591200	-0.07453800	-1.13100600	H	7.03967400	-2.31002700	-0.16895600
H	-7.29399800	-1.51144900	-0.27242900	H	6.37412000	-0.68333500	-0.11508300
H	-6.57421300	-0.17478600	0.61454800	H	6.19115400	-1.75422700	1.26742600
P	1.77714700	-1.79034300	-0.28711000	P	0.39689100	2.20196300	-0.22629100
O	1.33445000	-3.15206300	0.36454500	O	1.15121100	3.34078300	0.55943800
O	3.14169400	-2.17007300	-0.97819000	O	-0.56622400	3.01510600	-1.16644700
C	2.17051500	-3.86681300	1.28168800	C	2.06481200	3.02084600	1.61597600
H	2.44205700	-3.23250200	2.11823500	H	2.49797600	3.95798100	1.93867600
H	1.58605000	-4.70641000	1.63372800	H	1.53480300	2.55545500	2.43921200
H	3.06056000	-4.22328900	0.77606800	H	2.84605500	2.36209800	1.25242400
C	3.93194100	-1.17082800	-1.63324000	C	-1.47085900	4.00255300	-0.65669000
H	3.35863300	-0.69477700	-2.42112100	H	-2.05858700	3.59724000	0.15950200
H	4.26940800	-0.43119300	-0.91575400	H	-0.91809300	4.87310400	-0.32340900
H	4.78272200	-1.68554200	-2.05910600	H	-2.12163900	4.27036500	-1.47833600

DMHP-PC

O	0.39666400	-1.53900400	-0.08899900
C	1.25406600	-2.69900800	-0.11987400
H	1.15719800	-0.04555600	-0.80507000
H	0.97823100	-3.34385200	0.70708900
H	1.08146200	-3.23242600	-1.04790400
O	1.53032100	0.81624800	-1.06449200
O	-0.16821800	1.78751600	0.60948800
H	-1.08215300	0.18611500	0.28288800
N	-1.62277900	-0.64809600	0.08236100
C	-0.93369900	-1.79064500	-0.11005200
O	-1.37926800	-2.89854700	-0.27819000
C	-3.01592200	-0.48582300	0.09322800
C	-3.48971300	0.80127500	0.35033000
C	-3.92462300	-1.51560600	-0.13832200
C	-4.84906300	1.05322300	0.37666800
H	-2.78439400	1.59721100	0.52786200
C	-5.28541600	-1.24564600	-0.10865800
H	-3.57243300	-2.50948100	-0.33494500
C	-5.75931700	0.03072900	0.14724000
H	-5.19574400	2.05440100	0.57733500
H	-5.97889000	-2.05174900	-0.28902100
H	-6.81889200	0.22710500	0.16787400
C	2.68508600	-2.23200400	-0.00096500
H	2.93627900	-1.59882200	-0.85103000
H	2.79082600	-1.62557600	0.89795300
C	3.65058000	-3.41039600	0.05448700
H	3.52179900	-4.02461100	-0.83659500
H	3.39831300	-4.04241900	0.90565400
C	5.10133300	-2.96643100	0.16135200
H	5.77184500	-3.82220300	0.20158500
H	5.38986100	-2.35828900	-0.69443600
H	5.26491500	-2.37347700	1.05975800
P	1.13942000	1.95955500	-0.04834600
O	2.28483900	2.05784600	1.03602900
O	1.30084000	3.21143500	-0.99386600
C	1.03039100	4.52579800	-0.48876800

H	1.68959800	4.75853400	0.34090800
H	1.21674000	5.20902000	-1.30618400
H	-0.00453100	4.59982100	-0.17422900
C	3.66339400	2.17512900	0.66489600
H	3.98011900	1.29527800	0.11619600
H	3.82289000	3.06464300	0.06516300
H	4.22109600	2.25215000	1.58867500

**MSA**				**MSA-RC1**			
H	0.95344100	-1.81948300	-0.13944500	O	0.77905800	-1.13399600	0.32807200
O	0.76762500	-1.08225600	-0.74172800	C	1.76404100	-0.56570100	-0.51006300
S	0.08919800	0.12919200	0.05641200	H	-0.02106300	-1.25737000	-0.19098300
O	0.51821600	0.05525500	1.43370500	H	1.40187400	0.36427800	-0.95427100
O	0.37480600	1.30615700	-0.72240600	H	2.00383600	-1.24414600	-1.33211700
C	-1.62857100	-0.25321900	-0.05200600	C	3.00483200	-0.29480600	0.31136600
H	-1.90758900	-0.31111000	-1.09742800	H	2.74001300	0.36843400	1.13544600
H	-2.17326100	0.54566600	0.44000900	H	3.33900200	-1.23191600	0.75738500
H	-1.81351700	-1.19607400	0.44974300	C	4.13305000	0.32093600	-0.50538200
				H	3.78702200	1.25233700	-0.95473700
				H	4.38477100	-0.34327100	-1.33276600
				C	5.38004200	0.59182900	0.32304400
				H	6.16956100	1.03232800	-0.28259300
				H	5.16685100	1.27905700	1.14052000
				H	5.76964900	-0.32729200	0.75806800
				O	-1.84774800	-0.88638600	-0.83389000
				O	-3.17210700	-0.51038800	1.21328000
				O	-3.71991700	0.75889700	-0.77238100
				H	-2.59138400	-1.22162700	1.52849000
				S	-2.59424500	0.13857100	-0.12760000
				C	-1.47773900	1.36220600	0.47301400
				H	-0.66180600	0.86242600	0.98257200
				H	-1.10534000	1.91129600	-0.38554900
				H	-2.01471000	2.02652500	1.13961200

**MSA-RC2**				**MSA-TS**			
O	-1.50498600	-0.07992400	-0.68311200	O	1.58237800	0.11013700	-0.23461400
H	-0.76118800	-0.68509200	-0.75691500	C	2.89896600	-0.17138800	-0.73412000
O	0.57199000	-2.07279200	-0.63626800	H	1.22713800	0.92824300	-0.63149600
S	1.91558900	-2.43986500	-0.22768100	H	3.31023000	0.76182500	-1.10672900
O	2.24750700	-1.68038600	1.12528900	H	2.81090500	-0.86605200	-1.56425500
O	2.21524100	-3.82887000	0.00145200	O	0.34619700	2.36400200	-1.05955400
H	1.70541800	-0.86684200	1.22348300	S	-0.54607100	2.88949500	-0.02747400
N	0.72714500	0.71742300	1.36924800	O	-0.71912900	1.78537300	1.07295400
C	-0.35884100	0.51392400	1.86508500	O	-0.16695500	4.10748700	0.64118700
O	-1.32824200	0.22098200	2.42342300	H	-0.67704000	0.84330900	0.69110100
C	1.21343600	1.78251000	0.57989200	C	0.30762700	-1.24941000	-0.40512000
C	0.35341000	2.66925500	-0.05694100	C	-4.45192200	-2.57965100	0.26964800
C	2.58852500	1.91313100	0.44668700	C	-3.40756000	-3.20813700	-0.39124800
C	0.88215900	3.69670500	-0.81967100	C	-4.23623500	-1.34005400	0.85286300
H	-0.71199900	2.54047000	0.03185500	C	-2.99272800	-0.73556800	0.77633000
C	3.10517800	2.94450800	-0.32216800	C	-1.94367400	-1.36678600	0.11321300
H	3.24362900	1.22045900	0.94860700	C	-2.15917900	-2.61174500	-0.47360300
C	2.25618600	3.83942000	-0.95511800	H	-1.36305700	-3.11606400	-0.99317200

H	0.21501100	4.38529800	-1.31231300	H	-3.55979600	-4.17171300	-0.85056700
H	4.17350100	3.04671700	-0.42157100	H	-5.03661400	-0.83753600	1.37175700
H	2.66063800	4.64087500	-1.55135700	H	-2.83225100	0.22530200	1.23596900
C	3.07339100	-1.75677300	-1.36662500	N	-0.70387000	-0.67535000	0.07229000
H	4.07508200	-1.94916500	-1.00030100	O	0.86879300	-2.16061600	-0.87955600
H	2.92036300	-2.24664200	-2.32239700	H	-5.41993300	-3.04965900	0.32951700
H	2.89377600	-0.69160300	-1.45595500	C	3.74923700	-0.74518500	0.37432900
C	-2.68119800	-0.84990100	-0.54973100	H	3.79456900	-0.02675100	1.19186700
H	-2.63768700	-1.46572000	0.35074900	H	3.26714200	-1.64199700	0.76213900
H	-2.79086900	-1.52678300	-1.40016000	C	5.15607100	-1.08224300	-0.10365400
C	-3.87050500	0.08184800	-0.48015900	H	5.62438200	-0.18515800	-0.50930600
H	-3.88317900	0.69847800	-1.37931500	H	5.09684300	-1.79712000	-0.92451400
H	-3.73473800	0.75826600	0.36412000	C	6.02777800	-1.65267500	1.00475000
C	-5.19412500	-0.65840400	-0.34159000	H	7.02536800	-1.88749600	0.63946100
H	-5.17070900	-1.27539500	0.55722400	H	6.13273900	-0.94460900	1.82521000
H	-5.31401100	-1.34435100	-1.18084600	H	5.59869700	-2.56759500	1.41024500
C	-6.39047700	0.27951300	-0.28081800	C	-2.15379400	3.05809700	-0.72759200
H	-6.45966700	0.88594200	-1.18267500	H	-2.45121300	2.10660800	-1.15319300
H	-7.32244100	-0.27342500	-0.18117700	H	-2.84098000	3.36300800	0.05301700
H	-6.31528800	0.95790000	0.56776100	H	-2.10069700	3.81639900	-1.50161600

MSA-P

O	-1.02531900	-0.98082900	-0.00407700
C	-2.12955200	-1.87453700	0.26210300
H	-1.38217300	0.68977500	0.48051600
H	-1.93320800	-2.81015700	-0.24780900
H	-2.16968700	-2.06250800	1.32970300
O	-1.62503100	1.59812500	0.76241100
S	-0.71976900	2.67784700	0.03122800
O	-0.03265200	2.00430800	-1.05851600
O	-1.55795900	3.80864200	-0.26858800
H	0.79551300	0.21133700	-0.70388000
N	1.14282800	-0.62177800	-0.25332200
C	0.21223400	-1.49629000	0.18467600
O	0.39796500	-2.58044200	0.67525500
C	2.53914300	-0.75977200	-0.20158000
C	3.29104500	0.27280700	-0.76103500
C	3.19064300	-1.84733500	0.37403900
C	4.67277200	0.21810500	-0.74718400
H	2.78811700	1.11729800	-1.20526500
C	4.57752800	-1.88692900	0.38022100
H	2.62281300	-2.64802000	0.80645900
C	5.32783200	-0.86366800	-0.17586500
H	5.23654600	1.02580200	-1.18565100
H	5.07069400	-2.73472800	0.82857200
H	6.40465700	-0.90700800	-0.16528600
C	0.44598200	3.10990500	1.27962800
H	1.10117600	3.87056300	0.86850300
H	-0.09046100	3.49846700	2.13738500
H	1.01468800	2.22680600	1.54793400
C	-3.39498800	-1.22505100	-0.24446000
H	-3.56327100	-0.28387600	0.27727200

H	-3.27674100	-0.99156400	-1.30161400
C	-4.60039900	-2.13703400	-0.04625400
H	-4.69954900	-2.37958800	1.01171800
H	-4.42753200	-3.07966900	-0.56519900
C	-5.89172200	-1.50917500	-0.54762300
H	-6.73688200	-2.17736200	-0.39665900
H	-6.10572100	-0.57899300	-0.02379300
H	-5.83162600	-1.28425400	-1.61117400

TFMSA				TFMSA-RC1			
O	1.22592300	0.00175700	1.43348700	O	-1.34478400	-1.64088400	-0.57612300
S	0.83788000	-0.13527600	0.05946000	C	-2.11291100	-0.88667000	0.33695600
O	1.24651900	1.15320800	-0.75838200	H	-0.49487000	-1.81924300	-0.16959300
O	1.20220700	-1.26995000	-0.73123200	H	-1.57184900	0.00783600	0.65095100
H	1.36281700	1.92694300	-0.17794700	H	-2.32278500	-1.46903900	1.23776100
C	-0.98760200	0.00908100	-0.00246500	C	-3.41057600	-0.49105000	-0.33202300
F	-1.50623800	-0.98080400	0.68906900	H	-3.17763200	0.07778800	-1.23259000
F	-1.39511600	-0.05554700	-1.24863600	H	-3.92684900	-1.39523800	-0.65566800
F	-1.34758800	1.15889100	0.52516600	C	-4.31911900	0.32653200	0.57628400
				H	-3.78994500	1.22153800	0.90506700
				H	-4.54057600	-0.24671000	1.47710300
				C	-5.62133800	0.72878000	-0.09987600
				H	-6.25160100	1.31114500	0.56934500
				H	-5.43309900	1.33230400	-0.98659300
				H	-6.18874100	-0.14697100	-0.41130600
				O	1.38861300	-1.50548200	0.57224700
				O	3.03194900	-0.75151000	-1.11664100
				O	3.35437100	-0.07937700	1.19311100
				H	2.51328700	-1.36838000	-1.66440400
				S	2.33532900	-0.46530900	0.26895300
				C	1.37300600	1.03812400	-0.15120100
				F	0.72615400	1.42850800	0.92437800
				F	0.51958700	0.74968300	-1.10743600
				F	2.19024000	1.98443700	-0.54860100

TFMSA-RC2				TFMSA-TS			
O	-2.00400900	-0.16760500	-0.76478200	O	2.14758700	-0.29825400	0.41686200
C	-3.00944500	-1.15332400	-0.63476000	C	3.48373100	-0.22845800	0.89177600
H	-1.15556400	-0.61160000	-0.83198100	H	1.61771100	-0.83659300	1.01338500
H	-2.85380200	-1.74227800	0.27117300	H	3.75894400	-1.17955600	1.34581000
H	-2.97615900	-1.84273300	-1.48098300	H	3.56675000	0.54012700	1.66159100
O	0.56244600	-1.62206900	-0.62605300	O	-0.02739700	-1.87905700	1.23922800
S	1.67820200	-1.94278800	0.22688900	S	-0.57427300	-2.13797100	-0.08117100
O	1.63683200	-1.08486800	1.53193700	O	-0.41712300	-0.94983600	-1.00098300
O	1.97789500	-3.29660600	0.57951700	O	-0.23722800	-3.37460600	-0.73830400
H	1.02660000	-0.29926000	1.46458000	H	-0.16390200	0.14816400	-0.52245300
C	3.16860900	-1.25304700	-0.58333600	C	-2.38290200	-2.17635000	0.16459200
F	3.35150600	-1.88021500	-1.72403500	F	-2.69675900	-3.15187200	0.99544200
F	4.21358400	-1.42741100	0.19306100	F	-2.99467900	-2.37532100	-0.98675800
F	2.98885700	0.03076700	-0.80528500	F	-2.78948900	-1.02789300	0.67427100
N	-0.07727500	1.07139100	1.38587700	C	0.97065400	1.87141400	0.17481600
C	-1.17952400	0.78116100	1.80655400	C	-3.73739000	3.42138500	-0.19783500
O	-2.16125800	0.42067100	2.29453800	C	-2.78986800	3.72365400	0.76854800

C	0.33186700	2.17954700	0.60054700	C	-3.47657500	2.43456800	-1.13680700
C	-0.56063700	2.84990200	-0.22612300	C	-2.27040800	1.75240100	-1.12195700
C	1.66217700	2.56443600	0.66997200	C	-1.33482100	2.07086000	-0.15165500
C	-0.11110300	3.92408200	-0.97583800	C	-1.58305600	3.04362700	0.80275400
H	-1.58326300	2.51899600	-0.29429500	H	-0.85650800	3.26216200	1.56863700
C	2.09957300	3.63959700	-0.08748700	H	-2.98822800	4.48388500	1.50581100
H	2.34165100	2.03335400	1.31561500	H	-4.20947800	2.19344000	-1.88888200
C	1.21640200	4.32319200	-0.90927800	H	-2.05987400	0.99160500	-1.85377800
H	-0.80244400	4.44607700	-1.61725300	N	-0.09738200	1.32891300	-0.14899400
H	3.13272700	3.94121400	-0.03105100	O	1.89248400	2.48316800	0.45953700
H	1.55991600	5.15887600	-1.49671100	H	-4.67611300	3.95001400	-0.21680500
C	-4.35673400	-0.46871700	-0.58182300	C	4.40801100	0.08272400	-0.26435000
H	-4.36089900	0.22792500	0.25643200	H	4.30397400	-0.70146100	-1.01399400
H	-4.48108000	0.12548800	-1.48747400	H	4.08792400	1.01285600	-0.73425000
C	-5.51298900	-1.45032000	-0.44391500	C	5.86374600	0.19975300	0.16796700
H	-5.37494200	-2.04662100	0.45854800	H	6.17282800	-0.72818900	0.65006200
H	-5.49485700	-2.15022100	-1.27986000	H	5.95515600	0.98242500	0.92155600
C	-6.86765200	-0.75978400	-0.39120400	C	6.79924700	0.50252200	-0.99293300
H	-7.67615500	-1.48123500	-0.29105800	H	7.83157400	0.58151300	-0.65806700
H	-6.92681400	-0.07604800	0.45431400	H	6.75443100	-0.28118300	-1.74760000
H	-7.04924300	-0.18185400	-1.29614600	H	6.53441200	1.44178000	-1.47612900
TFMSA-PC							
O	-1.22165200	-1.29751700	0.00867100				
C	-2.39092200	-2.06169500	0.39794600				
H	-1.40742100	0.34653400	0.33305200				
H	-2.25844400	-3.07391000	0.03616100				
H	-2.44056700	-2.08057100	1.48097200				
O	-1.57313900	1.30843900	0.51940600				
S	-0.77324500	2.24194100	-0.44808200				
O	-0.21892600	1.46575500	-1.52887300				
O	-1.53541300	3.42738000	-0.69234500				
H	0.66653300	-0.43677800	-0.95630500				
C	0.62656000	2.70869100	0.63692700				
F	0.17851000	3.36028300	1.68480000				
F	1.44826700	3.47282500	-0.04749600				
F	1.25324600	1.61817400	1.02460400				
N	0.95981300	-1.19012100	-0.35734300				
C	-0.02076200	-1.88485700	0.25486300				
O	0.08922800	-2.87591900	0.92720400				
C	2.34551600	-1.41942500	-0.28878700				
C	3.16836700	-0.48276500	-0.91219200				
C	2.91536800	-2.51383300	0.35500100				
C	4.54222900	-0.64022100	-0.89513000				
H	2.72952600	0.36947300	-1.40677100				
C	4.29558300	-2.65497800	0.36688600				
H	2.29153900	-3.24158100	0.83638100				
C	5.11706000	-1.72823100	-0.25386100				
H	5.16276700	0.09377600	-1.38354400				
H	4.72632500	-3.50699100	0.86823300				
H	6.18771200	-1.85048800	-0.23932600				
C	-3.60680800	-1.40977900	-0.21491900				

H	-3.70986300	-0.39072700	0.15556500
H	-3.47348100	-1.35006200	-1.29402100
C	-4.87280900	-2.19479400	0.10966800
H	-4.98454500	-2.26853400	1.19127300
H	-4.76938700	-3.21416800	-0.26151900
C	-6.11797800	-1.55829200	-0.48828900
H	-7.00792700	-2.13444000	-0.24435900
H	-6.26229000	-0.54751900	-0.11034500
H	-6.04666400	-1.49978000	-1.57309100
